# The Influence of Plasma-Assisted Production and Milling Processes of DLC Flakes on Their Size, Composition and Chemical Structure

**DOI:** 10.3390/ma13051209

**Published:** 2020-03-08

**Authors:** Tomasz Kaźmierczak, Piotr Niedzielski, Witold Kaczorowski

**Affiliations:** Faculty of Mechanical Engineering, Institute of Material Sciences and Engineering, Lodz University of Technology, Stefanowskiego 1/15, 94-924 Lodz, Poland; kazmierczak.tomasz@gmail.com (T.K.); piotr.niedzielski@p.lodz.pl (P.N.)

**Keywords:** diamond-like carbon, flakes, plasma, milling, Raman spectroscopy

## Abstract

Diamond-like carbon (DLC) flakes were produced using a dual-frequency method: microwave/radiofrequency plasma-assisted chemical vapour deposition (MW/RF PACVD) with the use of methane or its mixture with gases such as hydrogen, argon, oxygen or nitrogen. Their modification was performed using a planetary ball mill with and without a fluid: deionised water or methanol. Changes occurring in the morphology of flake surfaces were presented in pictures taken using a scanning electron microscope (SEM). Their composition and chemical structure were analysed using Raman spectroscopy and X-ray photoelectron spectroscopy (XPS). The presented research results show that it is possible to control the size of flakes and their chemical structure. An increase in the C-C sp^3^ bond content in produced carbon-based materials is only possible by modifying DLC flakes during their production process by introducing oxygen or argon into the working chamber together with the carbon-carrying gas. In the processes of mechanical DLC flake modification, it is necessary to add fluid to limit the occurrence of graphitisation processes. The research conducted shows that methanol is best used for this purpose as its use results in a decrease in the percentage of C-C sp^3^ bonds as compared to the materials, before milling, of only 1.7%. A frequent problem both in the production of DLC flakes and during their mechanical modification is the introduction of additional elements into their structure. Admixing electrode materials from the plasma-chemical device (iron) or grinding beads (zirconium) to DLC flakes was observed in our studies. These processes can be limited by the appropriate selection of production conditions or by mechanical modifications.

## 1. Introduction

Carbon materials in a fragmented or thin-layered form are the basis for numerous contemporary studies and applications. The largest number of studies in this area, as presented in the global literature, pertain to powders in the form of nanotubes, graphene or nanodiamonds [1,2,3]. Excellent methods for their production, cleaning and functionalisation are known [4,5,6,7]. This, however, does not change the fact that this area is still valid and numerous researchers continue their studies on other forms of carbon, including diamond-like carbon (DLC). DLC is excellent as a low-friction or anti-wear coating [8,9] especially in medical applications [10,11] and it is often modified by adding various elements, e.g., Si, Ag, F or Ti [12,13,14]. It can also be used for the production of carbon-based fragmented materials, which are usually described in the literature as flakes or powders [15,16]. The name of DLC covers many types of materials which are mostly a mixture of C-C sp^3^ and C=C sp^2^ bonds and also hydrogen [17,18]. Raman spectroscopy and X-ray photoelectron spectroscopy (XPS) are usually used to identify these materials. The former allows qualitative determination of the chemical structure, in particular, the presence of characteristic bonds between carbon atoms as well a carbon and hydrogen. The obtained spectra, after deconvolution, are helpful in the specification of the ID/IG ratio, whose change may directly indicate changes in the number of C-C sp^3^ bonds in DLC. The other technique, i.e., XPS, allows a quantitative evaluation of both chemical composition and chemical structure of carbon materials.

Carbon powders, especially DLC flakes, are a very interesting material for numerous applications. We can find them, amongst other things, as an element of composites [15], material for biomedical applications [16,19,20], or in light-emitting diodes [21]. On the one hand, their use as a polymer matrix filler can improve the mechanical and tribological properties of composites. On the other hand, they are an interesting model material for examining the biological properties of carbon coatings. A number of reports can be found in the literature related to their impact on the cellular level, which show their low toxicity [16,22]. Like the coatings, DLC flakes or powders can also be susceptible to doping of various elements and compounds (e.g., carbon quantum dots), which makes them an excellent material for fabrication of high-efficiency LEDs [20]. DLC flakes can be obtained using many methods (e.g., microwave-assisted pyrolysis); however, they are much more often produced in the form of delaminated carbon coating. The latter method uses the phenomenon of coating cracking when the maximum internal stress levels are exceeded. This effect can be enhanced by using a base with the coefficient of thermal expansion divergent from the coefficient of thermal expansion of DLC coating e.g., an aluminium base (as in Ohana et al. study [15]). In this type of research, processes based on chemical vapour deposition (CVD) methods are usually used; in particular, ones which use radio, dual-frequency activated plasma microwave/radiofrequency (MW/RF), DC or pulsed-bias voltage and a methane, acetylene or toluene atmosphere [19,20,21,22]. Additionally, the number of carbon powders formed in CVD plasma can be determined by the selection of an appropriate working atmosphere e.g., the largest quantities are created when a large amount of hydrogen is introduced into the reactor in relation to the carbon-carrying gas [21]. However, DLC flakes acquired using CVD methods are usually characterised by a broad range of sizes. Attempts are made to reduce this range by additional mechanical processing—milling [23]. Additionally, milling offers a lot of opportunities to acquire and dope carbon materials, including nanomaterials.

It would appear that the area of DLC flake use produced by CVD methods could be extended if research performed in this area led to the formation of materials characterised by appropriate purity and uniform size. The aspects listed above are particularly important for biological research conducted on DLC flakes in which all changes in the chemical and phase composition influence the results obtained. Unfortunately, the most popular methods of DLC flake acquisition involve the risk of elements from electrode or modified media (during production processes) or utensil and bead materials (during milling processes) becoming spontaneously doped to the flakes [23]. The presented problems are the basis of the research conducted and presented in this publication.

## 2. Materials and Methods

### 2.1. Diamond-Like Carbon Flakes Deposition Process

DLC flakes were produced using a device based on the MW/RF plasma-assisted chemical vapour deposition (PACVD) method which was described in earlier publications [16,19,23]. This technique combines the advantages of microwave (2.45 GHz) and radio frequency plasma (13.56 MHz), allowing the control of degree of ionisation of the working atmosphere and the level of ion energy using RF bias [8,24]. The conducted work shows that, using this method, the deposition rate of the DLC coatings can be varied from 1 to 18 µm/h, which is close to the growth rate of carbon nanowalls [25]. Preliminary research on the possibility of producing DLC flakes using MW/RF plasma was conducted using methane and its mixtures with hydrogen, argon, oxygen and nitrogen and time of 120 min, in accordance with the parameters presented in Table 1. All the carbon flakes were deposited at RF and MW forwarded power equal to 500 W, under the negative self-bias of 500 V. The processes have proven that it is possible to produce delaminated DLC coatings on water-cooled RF electrodes and have shown, at the same time, the influence of gases introduced into the working chamber on the morphology, composition and chemical structure of powders obtained. All processes were implemented using the same energy conditions at a pressure ranging from 100 to 130 Pa. For milling processes, carbon flakes were produced in a pure methane atmosphere over a 200-min period. After finishing of plasmochemical processes, flakes were collected with a brush from the electrode.

### 2.2. Milling Process

Mechanical powder modification was conducted using a PM100 (Retsch GmbH, Haan, Germany) planetary ball mill. The milling was performed in a zirconium oxide (ZrO_2_) dish with a capacity of 50 mL using ZrO_2_ beads with a diameter of 1 mm. Each time, these processes were performed using the 20:1 bead weight to DLC flake ratio. The applied speed of the mill ranged from 300 to 600 rpm and the milling process duration was 10 h. Fluid in the form of deionised water or methanol (10 mL) was added to the dish (or not). All of the parameters of milling processes were selected on the basis of calculations performed using the Taguchi method [23].

### 2.3. Surface Characterisation

The morphology of the DLC flakes produced was analysed using Scanning Electron Microscopy SEM (Hitachi S-3000N, Kyoto, Japan). Increments of materials produced in CVD processes were selected so as to allow for comparisons between the processes. SEM images, after milling processes, were enlarged so as to allow an analysis of the sizes obtained after mechanical processing.

The composition and chemical structure were analysed using X-ray photoelectron spectroscopy (XPS). In this part of the study, the ESCALAB-210 system (VG Scientific, Fison, Glasgow, UK)—equipped with a non-monochromatic Al source (Ka = 1486.6 eV) operating at 14.5 kV and 20 mA—was used. For the tested DLC flakes, the C1s peak calibration was performed for the peak position amounting to 284.6 eV [26,27]. To obtain a more accurate analysis, the C1s peak was adapted using Gauss–Lorentz curves corresponding to sp^2^ C-C, sp^3^ C=C and C-N, C-O, C=O bonds, which maximum values equal to approximately 284.5 eV, 285.3 eV, 285.93 eV, 286.1 eV and 288 eV, respectively [27,28]. Additionally, the chemical structure of the powders produced was analysed using Confocal Raman Spectroscopy, the inVia device (Renishaw plc, Gloucestershire, UK)), operating at a wavelength of 532 nm. These tests were performed with a spectral resolution of 1 cm^−1^ at a range from 1000 to 1800 cm^−1^. The exposure time was 200 s. The obtained spectra were deconvoluted using a two-peak model with Peakfit software v.4.12.

## 3. Results and Discussion

### 3.1. DLC Flakes Produced Using the MW/RF PACVD Method

**Figure 1:**
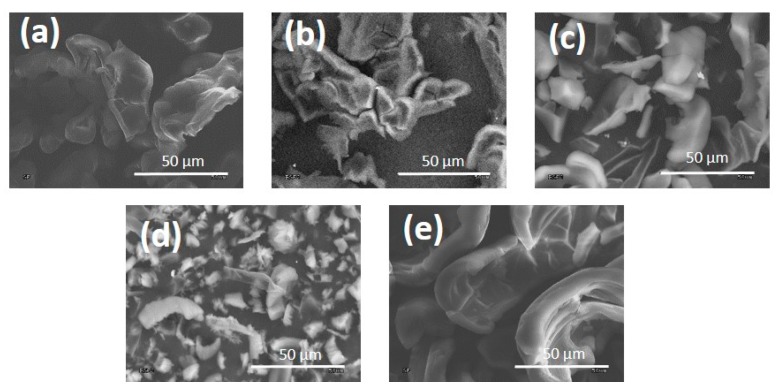
Powder produced using (**a**) methane; (**b**) methane and hydrogen mixture; (**c**) methane and argon mixture; (**d**) methane and oxygen mixture; and (**e**) methane and nitrogen mixture.

**Figure 2:**
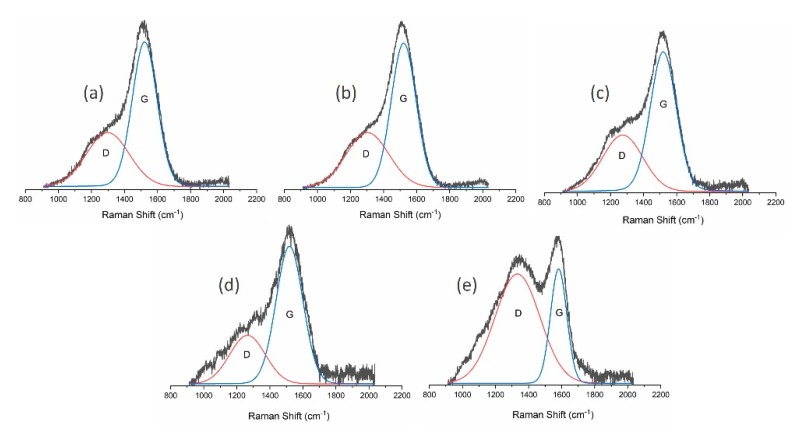
Raman spectra of powder produced using (**a**) methane; (**b**) methane and hydrogen mixture; (**c**) methane and argon mixture; (**d**) methane and oxygen mixture; and (**e**) methane and nitrogen mixture.

SEM pictures of the flakes produced in this part of the study are presented in Figure 1. The presented carbon materials were produced on the steel surface of an RF electrode using processes conducted in a methane atmosphere or its mixture with hydrogen, argon, oxygen and nitrogen (in accordance with Table 1). It can be seen in the pictures that the presence of oxygen and argon promotes the reduction of thickness and the size of the powders produced, probably due to the intensification of etching processes (Figure 2c,d). DLC flakes produced in an atmosphere of methane, methane with nitrogen or hydrogen have a shape of thick pieces of carbon coating detached from the electrode (Figure 2a,b,e). On the basis of the presented pictures, it can be established that the flakes produced in the methane plasma, or with the addition of hydrogen, have an average size of approximately 50 µm. When argon and oxygen were used as the additional gas, these parameters were reduced to 35 µm (for argon) –25 µm (for oxygen). Flakes produced in the methane-nitrogen plasma, on the other hand, had a size of over 80 µm.

Further analysis of the obtained carbon materials was conducted using Raman spectroscopy and XPS. Raman spectra of the DLC flakes produced are presented in Figure 2 and Table 2 contains a detailed analysis of them. It can be seen that a change of the gas mixture used in DLC flake production processes influences the obtained ID/IG ratio, which is closely correlated to the content of C-C sp^3^ bonds in produced flakes. The material obtained in the process conducted in the methane-oxygen atmosphere was characterised by a lowest ratio of 0.46, while the highest ID/IG parameters (2.64) were obtained for processes in the methane-nitrogen atmosphere. By analysing the parameters collected in Table 2, it can be concluded that powders produced with the use of methane-nitrogen plasma are graphite-like. The observed decrease in full width at half maximum of the G peak, together with an increase in its position value, is typical of graphite-like carbon materials (GLC) [29]. In case of other samples deposited by MW/RF PACVD method, the described parameters change is consistent with the changes in the ID/IG ratio.

**Figure 3:**
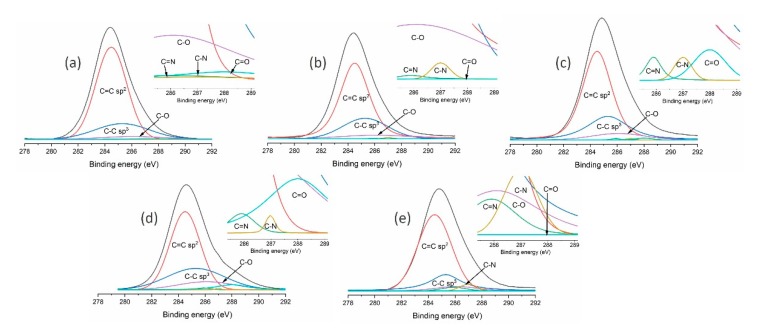
Analysis of C1 peaks obtained in X-ray photoelectron spectroscopy (XPS) studies of carbon flakes produced using (**a**) methane; (**b**) methane and hydrogen mixture; (**c**) methane and argon mixture; (**d**) methane and oxygen mixture; and (**e**) methane and nitrogen mixture.

An additional, more thorough analysis of the DLC flakes produced was performed using the XPS technique (VG Sci. ESCALAB-210, Fison, Glasgow, UK). The obtained structure and chemical composition of tested materials is presented in Table 3. The presented results were obtained by means of a characteristic peak analysis describing the C1 peak, as shown in Figure 3.

The presented results show that the chemical structure of the DLC flakes can be controlled by the working atmosphere. The highest content of C-C sp^3^ bonds may be obtained by introducing a methane-oxygen mixture or possibly a methane-argon mixture into the chamber. A decrease in the number of these bonds was observed for methane-hydrogen or, on the other hand, methane-nitrogen mixtures. The presence of oxygen in the flakes is a result of its adsorption on the carbon surface after the process deposition. Additionally, the level of oxygen in chemical composition depends on the fragmentation of the flakes. Additionally, the analysis of the DLC flake chemical composition revealed the presence of nitrogen and iron admixtures. Only subsequent work using a methane atmosphere showed that the extension of the preliminary vacuum chamber pumping time and the extension of the DLC flake production time (up to 200 min) allows DLC flakes to be produced without any admixtures (which will be shown in further studies). It can, therefore, be concluded that small quantities of nitrogen in the tested materials (except for the process in which nitrogen was intentionally introduced into the working mixture) can result from residual air present in the chamber. The admixture of iron, on the other hand, is related to plasma-related chemical processes (in particular etching) which occur on the steel high-frequency electrode during flake production. The intensity of these processes decreases as the electrode becomes covered by a carbon coating.

### 3.2. DLC Flake Milling

**Figure 4:**
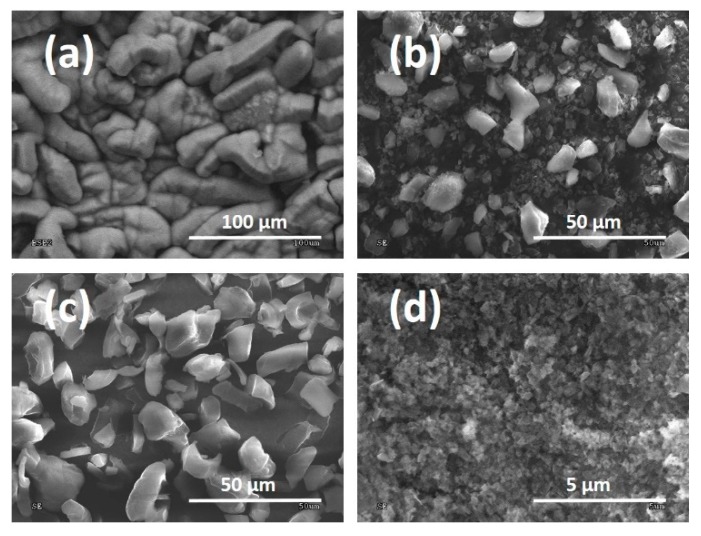
SEM pictures of powders: (**a**) produced using the chemical vapour deposition (CVD) method; (**b**) dry-milled; (**c**) milled in water; (**d**) milled in methanol.

Powders produced according to the procedure which guarantees the absence of admixture in the chemical composition (Process 1 in Table 1 with a production time of 200 min) were materials for milling in a planetary ball mill using (or not) using various fluids: deionised water or methanol, which is confirmed by XPS tests presented in this section. The influence of these processes on the obtained morphology of DLC flakes is presented in SEM pictures (Figure 4). Methane powders produced with the extended process duration had a different morphology than analogous ones produced over 120 min. Flake sizes obtained in these processes fell within a range of 50–100 μm. After milling without the addition of fluid, the flakes were fragmented but were characterised by high variability in size. Large pieces, which did not exceed 10 μm, could be seen in them but most had a size ranging from 0.5 to 2 μm. The addition of deionised water improved the uniformity of the powders obtained, whose size fell to within a range of 5–10 μm. If methanol was used, a good uniformity was also obtained in terms of size and the majority of powders fell within a range of 0.1–0.3 μm.

**Figure 5:**
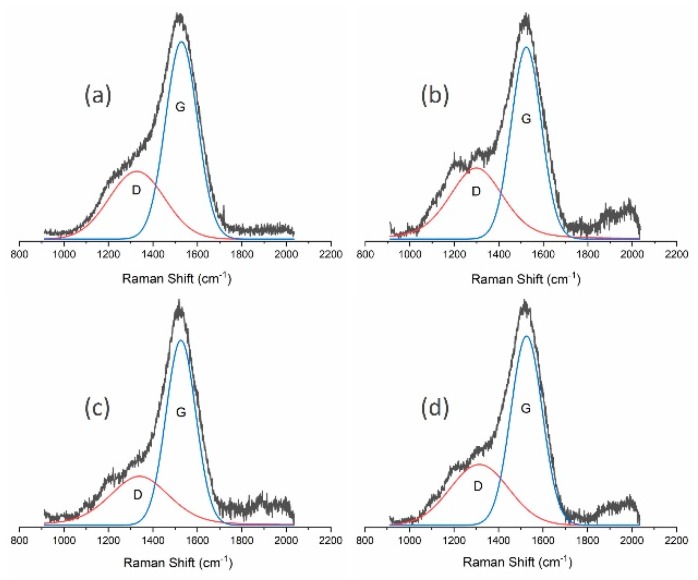
Raman spectra of powders: (**a**) produced using the CVD method; (**b**) dry-milled; (**c**) milled in water; (**d**) milled in methanol.

As in previous studies (and also in this case), an analysis was performed using Raman spectroscopy and XPS. Figure 5 presents spectra from Raman spectroscopy for unmodified powders and powders modified using the milling process, while Table 4 contains the most important parameters of the deconvoluted spectra.

**Figure 6:**
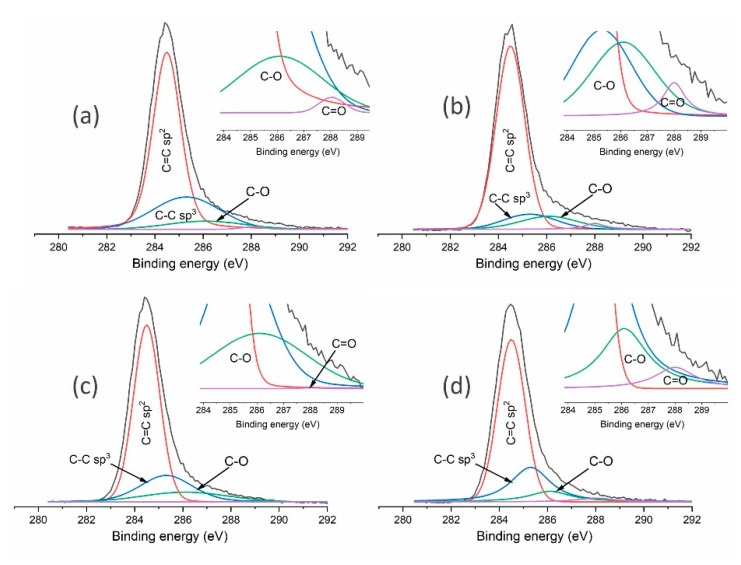
Analysis of C1s peaks obtained in XPS tests of powders produced using (**a**)the CVD method; (**b**) dry-milled; (**c**) milled in water; (**d**) milled in methanol.

The presented results from Raman spectroscopy prove the positive effect of introducing fluids into the milling process. As suggested by the obtained ID/IG ratios, they limit the graphitisation of powders. The ratio was 0.75 for dry milling, while its value for flakes before and after milling with the use of water or methanol was approximately 0.6. A more thorough analysis was conducted using the XPS method. The results of these tests for mechanically modified powders are presented in Table 5. As in the previous examples, the presented results were obtained by means of a characteristic peak analysis describing the C1 peak, as shown in Figure 6. XPS tests of the chemical structure after the milling processes show its changes. Dry-milling of powder leads to its graphitisation, which is shown by an increase in the percentage of C=C sp^2^ bonds (from 66.1% to 75.2%) and a decrease in the percentage of C-C sp^3^ bonds (from 25.9% to 11%). As a result, the sp^3^/(sp^3^ + sp^2^) ratio was also reduced from 0.28 to 0.13. This result is consistent with the Raman spectroscopy data, where an increase in the ID/IG ratio was observed for such powders. The limitation of the graphitisation phenomenon in powders, which occur as a result of their mechanical modification, can be seen if fluids are used for milling. The use of methanol proved to be the best in this respect. For these processes, the C-C sp^3^/(C-C sp^3^ + C=C sp^2^) ratio remained unchanged as compared to powders which were not milled, thus keeping the content of C-C sp^3^ bonds similar to the initial value; the ID/IG ratio from Raman spectroscopy tests was also comparable. A slight decrease in the C-C sp^3^ bonds was observed when water was used in the milling process (from 25.9% for the unmodified powder to 21.6%). XPS analysis of the obtained carbon flakes showed an increased amount of oxygen compared with the results for materials obtained during 120 min process of deposition. This effect is related to more extended surface area of deposited flakes. It was also determined, on the basis of XPS studies, that each type of mechanical modifications results in the introduction of zirconium into powders i.e., the material used in beads and the test dish. It can, therefore, be stated that there is a threat of polluting the obtained carbon materials. The most zirconium was found in powder milled in methanol (0.8%) and dry-milled (0.5%), while the use of water reduced this value to 0.1%.

## 4. Conclusions

This study presents the most important aspects of DLC flake production using the MW/RF PACVD method. The use of this method involves the risk of introducing additional elements from electrodes or media into the structure. However, this problem can be solved using longer processes of DLC flake production. At this stage of powder production, the chemical structure of a powder can also be controlled, for example, by the selection of a gas mixture added to these processes (such as in this study). However, one needs to be aware that the use of gases which allow for quicker etching of C=C sp^2^ bonds will involve a decrease in the efficiency of such processes. The addition of oxygen to the methane atmosphere had the greatest effect on the C-C sp^3^/(C-C sp^3^ + C=C sp^2^) ratio. It resulted in an increase in the content of C-C sp^3^ bonds in DLC flakes–from 24.3% to 28.8%. This effect is typical of the production of DLC coatings where the use of oxygen promotes the formation of C-C sp^3^ and the etching of the C=C sp^2^ bonds at the same time. A similar analogy for DLC coatings can be observed in studies using nitrogen and hydrogen whose addition can promote graphitisation of carbon materials. This can be clearly seen for nitrogen where the results obtained indicate a GLC structure. Additionally, the parameters of plasma-based processes influence the size of the flakes obtained. With the use of oxygen and argon as an additional gas introduced in the process (together with methane), sizes of approximately 25 and 35 μm were obtained respectively, i.e., approximately half less than for powders produced under different conditions.

When using the DLC flake milling process, one needs to remember, that just like in the production, about both the effect of introducing additional elements into the structure (Zr in the presented case) which come from dish walls and milling beads and the biggest hazard—the graphitisation of materials. Such mechanical processing causes an increase in the temperature which leads to the conversion of sp^3^ bonds into sp^2^. It is possible to limit this phenomenon by using fluid, which acts as an agent which reduces friction and takes away excess temperature from the system into the dish. It has been determined that the dry-milling processes cause a drastic decrease in the percentage of C-C sp^3^ bonds from approximately 26% (for the powder before milling) to 11%. In this case, an increase in the percentage of sp^2^ bonds is additionally observed (from 66.1% to 75.2%) and in carbon with oxygen. An analysis of the results for powders milled in water and in methanol makes it possible to establish that the fluids used can limit graphitisation processes which occur for dry milling. Methanol is the best in this respect as its use resulted in a decrease in the percentage of C-C sp^3^ bonds as compared to the materials before milling of only 1.7%. Additionally, an interesting result is the possibility of limiting the degree of pollution of produced carbon products by using water in the milling process. The presented results can be used for developing combined DLC flake production technologies with a strictly specified structure and chemical composition and high uniformity in size.

## Figures and Tables

**Table 1:** Parameters of carbon powder production processes.

RF Power [W]	MW Power [W]	Gas Flow [sccm]					Pressure [Pa]	Time of Deposition [min]
		CH_4_	H_2_	Ar	O_2_	N_2_		
500	500	180	-	-	-	-	100–130	120–200
			20	-	-	-		120
			-	20	-	-		120
			-	-	20	-		120
			-	-	-	20		120

**Table 2:** Analysis of Raman spectra of produced carbon flakes.

Sample	ID/IG	G Position (cm^−1^)	D Position (cm^−1^)	FWHM of G Peak (cm^−1^)
**CH_4_**	0.63	1521	1299	177
**CH_4_/H_2_**	0.65	1521	1303	177
**CH_4_/Ar**	0.60	1519	1274	186
**CH_4_/O_2_**	0.46	1519	1264	192
**CH_4_/N_2_**	2.64	1582	1333	115

**Table 3:** Analysis of chemical and phase composition of produced carbon flakes.

Sample	Structural Composition						Chemical Composition				sp^3^/ (sp^3^+sp^2^)
	C=C sp^2^ (284.5 eV)	C-C sp^3^ (285.3 eV)	C=N (285.9 eV)	C-O (286.1 eV)	C-N (287 eV)	C=O (288 eV)	C (%)	0 (%)	Fe (%)	N (%)	
**CH_4_**	68.1	24.3	0.4	5.8	0.4	1.0	92.8	6.3	0.3	0.6	0.26
**CH_4_/H_2_**	68.9	24.0	0.1	6.6	0.3	0.1	92.6	6.9	0.2	0.3	0.25
**CH_4_/Ar**	64.6	27.1	0.3	7.3	0.3	0.4	91.5	7.9	0.2	0.4	0.29
**CH_4_/O_2_**	54.6	28.8	0.6	10.7	0.2	5.1	82.1	16.9	0.3	0.7	0.35
**CH_4_/N_2_**	67.7	20.5	2.7	6.0	3.0	0.1	87.8	6.1	0.1	6.0	0.23

**Table 4:** Analysis of Raman spectra of powders produced using the CVD method, dry-milled, milled in water, milled in methanol.

Sample	ID/IG	G Position (cm^−1^)	D Position (cm^−1^)	FWHM of G Peak (cm^−1^)
**MW/RF PACVD**	0.60	1528	1327	169
**MW/RF PACVD after dry milling**	0.75	1523	1299	159
**MW/RF PACVD after milling with water**	0.61	1526	1340	158
**MW/RF PACVD after milling with methanol**	0.60	1526	1314	166

**Table 5:** Analysis of chemical and phase composition of powders produced using the CVD method, dry-milled, milled in water, milled in methanol.

Sample	Structural Composition				Chemical Composition			sp^3^/(sp^3^ + sp^2^)
	C=C sp^2^ (284.5 eV)	C-C sp^3^ (285.3 eV)	C-O (286.1 eV)	C=O (288 eV)	C (%)	0 (%)	Zr (%)	
**MW/RF PACVD**	66.1	25.9	7.5	0.6	90.5	9.5	-	0.28
**MW/RF PACVD after dry milling**	75.2	11.0	11.3	2.5	85.7	13.8	0.5	0.13
**MW/RF PACVD after milling with water**	66.8	21.6	11.6	-	88.3	11.6	0.1	0.24
**MW/RF PACVD after milling with methanol**	62.1	24.2	11.1	2.6	87.4	12.8	0.8	0.28
